# The Role of Light and Circadian Clock in Regulation of Leaf Senescence

**DOI:** 10.3389/fpls.2021.669170

**Published:** 2021-04-12

**Authors:** Juhyeon Lee, Myeong Hoon Kang, Jung Yeon Kim, Pyung Ok Lim

**Affiliations:** Department of New Biology, DGIST, Daegu, South Korea

**Keywords:** leaf senescence, light, PIFs, circadian clock, plant hormones

## Abstract

Leaf senescence is an integrated response of the cells to develop age information and various environmental signals. Thus, some of the genes involved in the response to environmental changes are expected to regulate leaf senescence. Light acts not only as the primary source of energy for photosynthesis but also as an essential environmental cue that directly control plant growth and development including leaf senescence. The molecular mechanisms linking light signaling to leaf senescence have recently emerged, exploring the role of Phytochrome-Interacting Factors (PIFs) as a central player leading to diverse senescence responses, senescence-promoting gene regulatory networks (GRNs) involving PIFs, and structural features of transcription modules in GRNs. The circadian clock is an endogenous time-keeping system for the adaptation of organisms to changing environmental signals and coordinates developmental events throughout the life of the plant. Circadian rhythms can be reset by environmental signals, such as light-dark or temperature cycles, to match the environmental cycle. Research advances have led to the discovery of the role of core clock components as senescence regulators and their underlying signaling pathways, as well as the age-dependent shortening of the circadian clock period. These discoveries highlight the close relationship between the circadian system and leaf senescence. Key issues remain to be elucidated, including the effect of light on leaf senescence in relation to the circadian clock, and the identification of key molecules linking aging, light, and the circadian clock, and integration mechanisms of various senescence-affecting signals at the multi-regulation levels in dynamics point of view.

## Introduction

Leaves are crucial to plant growth and survival. During early development, emerged leaves become photosynthetic organs that convert light energy into nutrients that are necessary for plant growth. Leaves in which photosynthesis is no longer productive begin to senesce. During leaf senescence, cellular constituents generated during the growth phase of leaves are converted into mobilizable nutrients and relocated to other developing organs. Thus, leaf senescence needs to be precisely tuned to ensure plant fitness (Woo et al., 2019).

Leaf senescence is an integral part of development despite the associated degenerative physiological changes. Under favorable conditions, this process occurs at age-dependent manner by an innate developmental program. However, unfavorable conditions, such as darkness or abiotic and biotic stresses, can induce premature leaf senescence, which shortens the lifespan of individual leaves. Sacrificing the inefficient and senescing organs would be beneficial for plants by making resources available to other organs. Besides its role in nutrient recycling, leaf senescence evolved as an adaptive strategy to respond appropriately to environmental changes (Schippers et al., 2015).

Light is a critical environmental factor affecting plant development. The importance of light for leaf senescence is becoming increasingly apparent. The regulatory networks linking light information and leaf senescence have been elucidated (Sakuraba et al., 2014; Song et al., 2014).

Plants are constantly exposed to environmental changes. To regulate development efficiently, plants have developed a circadian clock system. The circadian system is an endogenous time-keeping mechanism that measures daily and seasonal changes in the environment and allows plants to adjust physiological and developmental processes accordingly (Harmer, 2009). The circadian rhythm is entrained to cyclic environmental signals and can be reset by a variety of stimuli such as light signal. Thus, the circadian clock integrates environmental signals and coordinates developmental events throughout the life of the plant. Emerging evidence suggests that circadian clock core components are involved in leaf senescence.

Here, we review recent findings on leaf senescence, particularly the role of light and circadian clock, and how the senescence-regulatory networks are interacting with these signals. We also discuss the temporal and light-mediated regulation of plant physiology in relation to leaf senescence, which will contribute to understand the fitness and adaptive advantage of higher plants.

## Light as a Modulator of Leaf Senescence

Light perception by photoreceptors and light signaling components are crucial for modulating leaf senescence. Light-dependent retardation of leaf senescence is a low-fluence response that shows red (R)/far-red (FR) light reversibility. The R:FR ratio also affects leaf senescence: a low R:FR ratio induces leaf senescence, whereas a high R:FR ratio can delay it. These effects are of considerable ecological significance. Light passing through the upper leaves under field conditions contains reduced R relative to FR, and this FR-enriched condition might serve as a signal to trigger senescence of the lower leaves, which permits the allocation of resources to the upper shoot.

These responses are regulated by type II phytochromes, of which phytochrome B (phyB) is the main photoreceptor mediating R-induced senescence suppression in *Arabidopsis* (Reed et al., 1994). The mutation of *phyB* induced hyposensitivity to the continuous or pulsed R in retarding leaf senescence (Sakuraba et al., 2014). Continuous FR or low light also causes a substantial delay in leaf senescence compared with the effect of darkness, and these responses are modulated by phyA (Brouwer et al., 2014).

The light signals perceived by photoreceptors are transduced to the regulatory network that drives multiple facets of plant development including leaf senescence. Phytochrome-interacting factors (PIFs) interact with light-activated phytochromes, which inhibits their activities through several mechanisms; (1) phyB-PIF interaction leads to repress the DNA-binding ability of PIFs (Park et al., 2018), (2) it also results in phosphorylation and degradation of PIFs (Pham et al., 2018), and (3) PIFs are transcriptionally controlled, allowing PIF accumulation during the day time, even when phyB is active (Sun et al., 2019; Yan et al., 2020). Multiple mechanisms might be evolved for optimal light regulation of PIFs across a wide range of light conditions.

Among PIFs, PIF4, and PIF5 (PIF4/PIF5) are critical transcription factors (TFs) that mediate the induction of leaf senescence not only under dark conditions, but also under natural senescence conditions. In *Arabidopsis*, PIF4/PIF5 are upregulated at the early stage of leaf senescence as well as in response to darkness (Song et al., 2014). PIF4/PIF5 mutants display delayed leaf senescence under prolonged darkness and in response to long-day conditions. The increase of PIF4/PIF5 under dark conditions is inhibited by intermittent pulses of R, but not when pulses of R are followed by FR, indicating that active phytochromes prevent premature senescence in the presence of light by repressing PIF4/PIF5 expression (Sakuraba et al., 2014).

In recent years, much of the research on hormone signaling has focused on understanding the interplay between hormones and environmental signals including light and temperature, highlighting the importance of signaling (Jaillais and Chory, 2010). PIFs play key roles in integrating light and hormone signals through their function as TFs targeting genes involved in hormone biosynthesis or signaling and/or by interacting with components of hormone pathways.

Comparative transcriptome analysis of dark-induced senescence in *pif4* or quadruple *pif (pifQ)* mutants identified the subset of genes responsible for the PIF-mediated leaf senescence response. These include genes involved in chloroplast maintenance/photosynthesis, degradation of chlorophyll, responses to stresses/reactive oxygen species (ROS), and those involved in ethylene and abscisic acid (ABA) senescence-promoting signals, whose expressions are altered in *pif* mutants. Chromatin immunoprecipitation assays identified ABA-INSENSITIVE5 (ABI5), ENHANCED EM LEVEL (EEL), and ETHYLENE-INSENSITIVE3 (EIN3) as the direct target genes of PIF4/PIF5 (Sakuraba et al., 2014).

**Figure 1:**
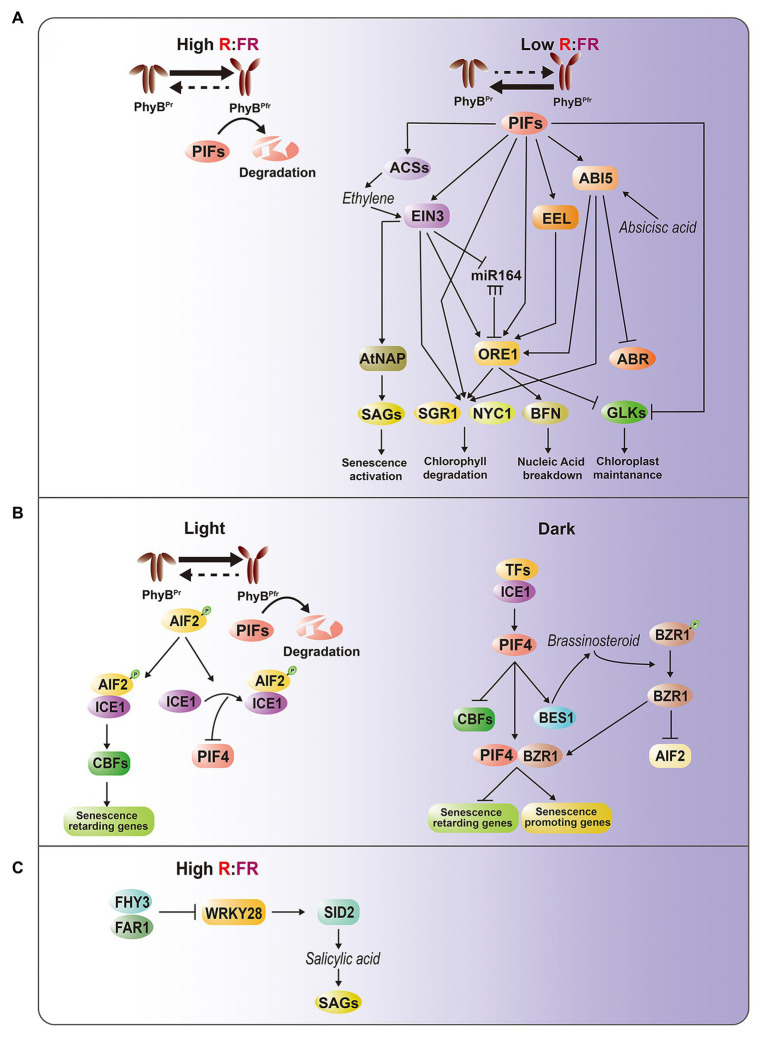
Intricate regulatory interactions between light and hormone signaling pathways in leaf senescence. **(A)** PhyB-PIFs-mediated senescence regulatory pathways. Under light or high R/FR condition, PIFs are degraded at a phyB-dependent manner, thereby suppressing PIF-dependent senescence activation. Under darkness, shade or low R/FR condition, phyB-mediated PIF degradation is inhibited, leading to activation of senescence-promoting ABA and ethylene hormone pathways. Direct targets of PIFs include EIN3 for ethylene signaling, and ABI5 and EEL for ABA signaling. Moreover, PIFs, EIN3, ABI5, and EEL activate *ORE1*, one of master regulators in leaf senescence, through binding to its promoter. Accumulating ORE1, PIFs, ABI5, and EIN3 subsequently activate *SGR1* and *NYC1* for chlorophyll degradation, whereas ORE1 alone induces *BFN* for nucleic acid degradation and other SAGs. On the other hand, PIFs and ORE1 repress the chloroplast maintenance master regulator GOLDEN2-LIKE (GLK) by suppressing its promoter activity and sequestrating protein through protein-protein interaction, respectively. EIN3 also regulates *ORE1* through *miRNA164*, and directly activates *AtNAP*, another senescence master regulator. ABI5 suppresses *ABR*. **(B)** AIF2-ICE1-mediated retardation of dark-induced senescence. PIFs are also involved in dark- and BR-induced leaf senescence at phyB-independent manner. In light, AIF2 interacts with ICE1 to directly downregulate *PIF4* and upregulate *CBFs*, which promote self-maintenance or senescence-repressing genes. In darkness, PIF4 promotes BR synthesis and *BZR1* activation, leading to decrease of AIF2. Activated BZR and PIF4 complex suppresses senescence-retarding genes and activates senescence-promoting genes. **(C)** FHY3-WRKY28 transcription module involved in interlinking between light and salicylic acid pathways. High R/FR condition activates *FHY3* and *FAR1*, subsequently suppressing *WRKY28*, which promotes SA biosynthesis.

*ABI5* and *EEL* encode basic leucine zipper (bZIP) TFs involved in ABA signaling, and the mutations of these genes cause delayed leaf senescence, suggesting that they are positive regulators of dark-induced leaf senescence (Sakuraba et al., 2014). ABI5 is also known to suppress ABA-response protein 5, a negative regulator of dark-induced leaf senescence (Su et al., 2016). EIN3 is a key TF involved in the EIN2-mediated ethylene signaling cascade that regulates age- and dark-induced leaf senescence by inducing two NAM, AFAT, and CUC (NAC) TFs, ORESARA1 (ORE1) and NAC-LIKE, ACTIVATED BY AP3/PI (AtNAP), which are the master regulators of leaf senescence (Kim et al., 2014). EIN3 also causes the accumulation of *ORE1* transcripts by directly repressing *microRNA (miR)-164* transcription (Kim et al., 2009). In addition to alterations of signal transduction, *pif4* mutants also show attenuated induction of ethylene biosynthesis by darkness (Song et al., 2014). Taken together, these results suggest that PIFs play an important role in transducing light information to ABA and ethylene pathways, thereby activating leaf senescence responses (Figure 1A).

bZIP- and EIN3-activated downstream genes are also markedly downregulated in *pifQ* mutants. Intriguingly, *ORE1* is a common direct target of PIFs, EIN3, EEL, and ABI5. Upregulation of *ORE1* activates genes involved in nucleic acid degradation, such as BIFUNCTIONAL NUCLEASE 1, as well as genes for chlorophyll catabolism, such as STAYGREEN 1 (SGR1/NYE) and NON-YELLOW COLORING 1 (NYC1; Song et al., 2014). *SGR* and *NYC1* are also direct targets of PIFs, EIN3, and ABI5 (Sakuraba et al., 2014; Song et al., 2014). ORE1 sequesters the chloroplast maintenance master regulators GOLDEN2-LIKE (GLK) 1 and GLK2 through protein-protein interactions, which decreases the transcriptional activity of GLKs during leaf aging (Rauf et al., 2013). At the transcriptional level, PIF4 acts as a repressor of *GLKs* (Song et al., 2014).

These findings suggest that the intricate gene regulatory networks (GRNs) governed by PIF4, PIF5, EIN3, EEL, ABI5, and ORE1 are linked, thereby forming multiple coherent feed-forward loops. These results also explain how the GRN modules involving PIFs coordinate various endogenous and environmental signals during leaf senescence.

Brassinosteroids (BRs) are senescence-accelerating hormones. ATBS1-INTERACTING FACTOR 2 (AIF2) was recently identified as a negative regulator of dark- and BR-induced leaf senescence in *Arabidopsis* (Kim et al., 2020). Molecular and genetic evidence led to the construction of a model describing the role of the interaction of light and BR signaling in the regulation of senescence (Figure 1B). BRASSINAZOLE-RESISTANT 1 family proteins (BZRs) are TFs that govern BR-regulated gene expression. Under conditions of darkness, PIF4 promotes BR synthesis and BZR1 activation, leading to a decrease of AIF2. As dark incubation proceeds, accumulated PIF4 together with BZR1 suppress senescence-retarding genes, such as C-REPEAT BINDING FACTORs (CBFs), and induces the expression of senescence-promoting genes, such as those involved in ethylene/jasmonic acid (JA) biosynthesis, and activates the corresponding signaling pathways. When leaves are exposed to light, accumulated AIF2 interacts with INDUCER OF CBF EXPRESSION 1 (ICE1). The AIF2-ICE1 complex and the subsequent upregulation of *CBFs* negatively regulate darkness-induced leaf senescence. This interaction also decreases *PIF4* transcription through the AIF2-dependent inhibition of ICE1 binding to the *PIF4* promoter, leading to the suppression of leaf senescence.

Recent evidence indicates that phytochrome-associated senescence regulation is interlinked with salicylic acid (SA) pathways (Tian et al., 2020). The *Arabidopsis* FAR-RED ELONGATED HYPOCOTYL 3 (FHY3) and its closest homolog FAR-RED IMPAIRED RESPONSE 1 (FAR1) play key roles in the phyA-mediated FR light signaling pathway (Lin et al., 2007). Disruption of *FHY3* leads to early leaf senescence in an age-dependent manner, as well as under high R:FR conditions, indicating that *FHY3* is a key negative regulator of age- and light-mediated leaf senescence. In addition, FHY3 represses the transcription of *WRKY28*, which promotes SA biosynthesis by activating SA INDUCTION DEFICIENT 2 (SID2; van Verk et al., 2011). The early senescence phenotype of the *fhy3* mutant is rescued by disruption of *WRKY28*, confirming the role of the *FHY3-WRKY28-SID2* transcriptional module in the regulation of leaf senescence. Given that FHY3 and FAR1 are important TFs involved in phyA-mediated FR light signaling (Lin et al., 2007), understanding the relationships among FHY3, phyA, and phyB may shed light on the regulatory mechanisms by which a high R:FR ratio inhibits leaf senescence (Figure 1C).

Further systematic studies are necessary to elucidate the detailed molecular mechanisms underlying the connections between light signaling pathways and other internal or external senescence-regulating programs. Identification of upstream regulators, downstream targets, and interaction molecules of light signal-associated TFs, such as PIFs or FHY3, at senescence conditions will help to dissect such intricate regulatory pathways of leaf senescence.

## Leaf Senescence and Circadian Clock

The timing of developmental transitions is critical for plant fitness. Plants may possess mechanisms to measure the passage of time, although a clear understanding of these processes in plants is lacking. The circadian clock is a ubiquitous endogenous time-keeping system that generates 24 h rhythms adapted to the light–dark cycle, and it predicts daily and seasonal changes in the environment (McClung, 2006). This endogenous clock regulates many aspects of development and physiology throughout the life of a plant, and it may be critical for the temporal coordination of development.

**Figure 2:**
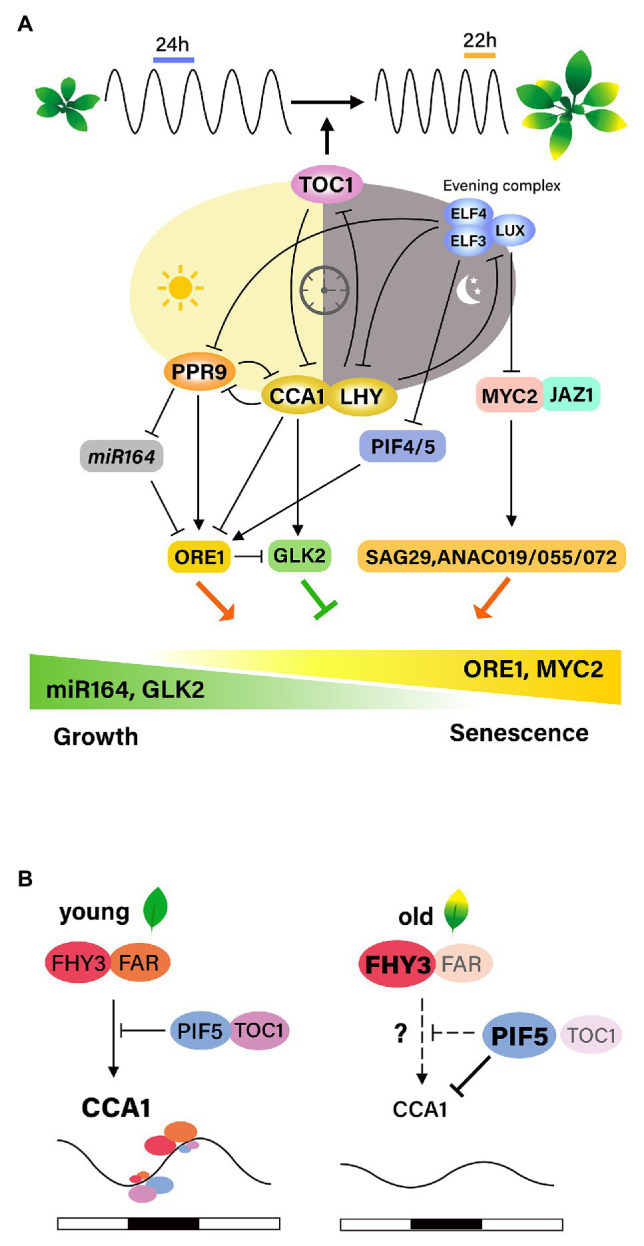
Interactions between the circadian clock and leaf senescence. **(A)** Age information integrates with the circadian clock through TOC1, one of the core oscillators, resulting in the shortening of the circadian period with leaf senescence. Age-declined CCA1 induced the expression of *ORE1* and suppression of *GLK2* along leaf aging. PRR9 in the morning loop directly activates ORE1, and indirectly suppress miRNA164, forming the feed-forward pathway. The evening complex (EC) negatively regulates jasmonate-induced leaf senescence by repressing the expression of *MYC2*. Inactivation of EC complex causes the activation of MYC2, which induces *SAG29*, *ANAC019*, *ANAC055*, and *ANAC072*, promoting leaf senescence. ELF3, a component of EC, negatively affects *ORE1* expression by repressing PIF4/5. **(B)** FHY3/FAR1 is required for the light-induced expression of CCA1 and the role of FHY3/FAR1 in activating CCA1 expression is antagonistically regulated by TOC1 and PIF5. Under diurnal cycle conditions, the high levels of FHY3 and FAR1 and low levels of PIF5 and TOC1 at the end of the evening contribute to the peak expression of the morning gene *CCA1* before dawn. However, at aged leaves, FHY3 plays a role as a negative regulator of leaf senescence by suppressing WRKY28. Age-declined CCA1 expression might be due to PIF5, whose activity is increased at senescence conditions.

In *Arabidopsis*, the core oscillator of the circadian clock consists of interlocking negative feedback loops. CIRCADIAN CLOCK-ASSOCIATED 1 (CCA1), LATE ELONGATED HYPOCOTYL (LHY), PSEUDO-RESPONSE REGULATOR 7 (PRR7), and PRR9 form a morning loop, whereas TIMING OF CAB EXPRESSION 1 (TOC1), EARLY FLOWERING 3 (ELF3), ELF4, and LUX ARRYTHMO (LUX) form an evening loop. ELF3, ELF4, and LUX are functionally associated and are components of the evening complex (EC). The morning and evening loops are interconnected and generate circadian outputs through transcriptional and post-transcriptional mechanisms, thereby enabling plants to express numerous genes at the proper time and phase (McClung, 2006). The relationship between senescence and the circadian clock, and the potential molecular mechanisms underlying their interaction have been explored recently (Wang et al., 2018; Figure 2).

The first hint comes from results showing that the circadian periods of clock-regulated genes as well as the periods of the core clock genes are shorter in older leaves than in young leaves of *Arabidopsis*. Such age-dependent period shortening is not observed in the disruption of TOC1, suggesting that TOC1 may link age to changes in the circadian clock period (Kim et al., 2016). These findings suggest that age-associated information is integrated into the regulation of the circadian period, and that TOC1 is necessary for this integrative process.

The involvement of circadian components in the regulation of leaf senescence has been analyzed in detail. Song et al. (2018) proposed that CCA1 regulates leaf senescence negatively based on findings that age-declined CCA1 upregulates *ORE1* and downregulates *GLK2* by binding to their promoters, thereby promoting the onset of leaf senescence. CCA1 functions as a master regulator of ROS homeostasis by interacting with the EC in promoters of ROS-responsive genes *in vivo*, and ROS function as an input signal that affects the transcriptional output of the clock (Lai et al., 2012). Therefore, identifying proteins that interact with CCA1 during leaf senescence, as well as downstream targets of CCA1, including ROS homeostasis-related genes, may help to elucidate the relationships between aging, ROS, the circadian clock, and leaf senescence.

Recent discovery showing that FHY3 and FAR1, phytochrome signal transducers, are necessary for the light-induced CCA1 expression is particularly illuminating. FHY3 and FAR1 activate *CCA1* expression through direct binding to its promoter, but dark-activated PIF5 suppresses the transcription of *CCA1* (Liu et al., 2020). Under diurnal condition, the role of FHY3/FAR1 in activating *CCA1* transcription is antagonistically controlled through interaction with TOC1 and PIF5, leading to daily oscillation of the *CCA1* expression. Taking previous findings on the roles of these components as regulators of leaf senescence, it is plausible that the proposed mechanism might be responsible for the effect of light on leaf senescence in relation with circadian clock. However, higher level of FHY3 at aged leaves does not explain age-declined CCA1. It is likely that during senescence. Increased activity of PIF5 might cause the suppression of CCA1 transcription (Figure 2B).

Extensive genetic analyses using core clock component mutants have been performed to identify senescence regulators (Kim et al., 2018). The EC components ELF3, ELF4, and LUX, as well as the morning component PRR9, affect leaf senescence. Mutation of PRR9 delays age-dependent, as well as dark-induced, leaf senescence. This may be mediated by the downregulation of leaf senescence regulators, such as *NAC* and *WRKY* TFs, with a circadian expression pattern. PRR9 directly promotes rhythmic transcription of *ORE1* by binding to the *ORE1* promoter and indirectly by suppressing the clock-controlled *miR-164*, a post-transcriptional repressor of *ORE1*, thus forming a coherent feed forward regulatory loop. Importantly, ORE1 overexpression restores age-associated senescence in *prr9* mutant plants. These results suggest that the circadian clock controls leaf senescence by modulating ORE1 amplitude by PRR9, suggesting an intimate relationship between leaf senescence and the circadian clock as shown in animals.

The molecular mechanism by which the core components of the circadian clock gate JA signaling to regulate leaf senescence was elucidated in *Arabidopsis* (Zhang et al., 2018). Mutations in EC components result in accelerated age-induced senescence, as well as more pronounced JA-induced leaf senescence. Global gene expression analyses indicate that the EC is associated with JA signaling and response pathways and also controls senescence regulators such as *WRKY53*, *WRKY70*, *ORE1*, and *AtNAP*. These results indicate that the EC may function as a negative component of the leaf senescence regulatory network by repressing senescence regulators. LUX binds directly to the promoter of *MYC2*, a JA downstream TF, and likely gates its JA-induced expression profile. Genetic analysis further demonstrated that the accelerated JA-induced leaf senescence in EC mutants is abrogated by the *myc2 myc3 myc4* triple mutation, confirming that a core component of the circadian clock gates JA signaling *via* MYC TFs to regulate leaf senescence (Zhang et al., 2018). On the other hand, ELF3 regulates dark-induced leaf senescence by repressing PIF4/PIF5 in an EC-independent manner, as ELF4 and LUX mutants do not show the accelerated senescence phenotype observed in ELF3 mutants under dark conditions (Sakuraba et al., 2014). Thus, it is plausible that intricate interactions of core components and diverse senescence responses are involved in the regulation of leaf senescence. The interaction networks between the circadian clock and leaf senescence need to be further elucidated.

## Perspectives and Future Challenges

Leaf senescence is a time-dependent developmental event. However, it also involves intricate interactions with various endogenous and exogenous signals. How the senescence-regulatory networks interact with these signals and how the interaction affects life and senescence in plants are long-standing questions. PIFs, which were initially recognized as components of the phytochrome signaling pathway, are currently considered as key players at the convergent points of light signals and internal hormone responses, which together modulate leaf senescence. PIFs and downstream regulators involved in senescence-promoting hormone signaling or synthesis control ORE1, a master regulator of leaf senescence. A picture is just beginning to emerge, and a comprehensive understanding of leaf senescence may require new approaches. For example, analyses to find direct targets of PIFs and their inter-players have been performed at specific developmental stages, mostly seedling, or under specific environmental conditions, which do not reflect the complete regulatory pattern of leaf senescence. Genome-wide analysis of PIF target genes or interacting molecules at leaf senescence stages is necessary.

The mechanism underlying the effect of light on plant senescence in relation to the circadian clock has also been long-term interest. PIF4/PIF5 are expressed rhythmically during the diurnal cycle, and their expression is regulated at the transcriptional and post-translational levels (Shin et al., 2013). PIFs are connected with various hormone responses, which are additionally regulated by the circadian clock as output pathways. However, whether PIFs affect the clock function in a light-dependent manner remains unclear, and, if so, the molecular mechanism by which PIFs transduce light information to the core oscillator during leaf senescence needs to be elucidated.

Analysis of PIF- or circadian core component-mediated regulation of leaf senescence has mainly focused on transcriptional regulation, which revealed the importance of multiple feed-forward loops in the regulation of leaf senescence. To better understand leaf senescence, it is necessary to perform multilayered interaction-based analyses of senescence. These should include the dynamics of PIF-interactomes (protein-protein, protein-DNA, protein-RNA, and RNA-RNA) complexes in a developmental time-dependent manner or spatial networks that involve organellar interactions.

The link between the circadian clock and leaf senescence is clear. However, the association of leaf senescence with changes in the circadian system remains unclear, particularly the potential causal relationship between them. The complex underlying regulatory network needs to be fully elucidated.

## Author Contributions

JL, MK, JK, and PL reviewed literature and participated in writing the manuscript. All authors contributed to the article and approved the submitted version.

### Conflict of Interest

The authors declare that the research was conducted in the absence of any commercial or financial relationships that could be construed as a potential conflict of interest.
